# Development and characterization of a human three-dimensional chondrosarcoma culture for *in vitro* drug testing

**DOI:** 10.1371/journal.pone.0181340

**Published:** 2017-07-13

**Authors:** Aurélien Voissiere, Elodie Jouberton, Elise Maubert, Françoise Degoul, Caroline Peyrode, Jean-Michel Chezal, Élisabeth Miot-Noirault

**Affiliations:** Université Clermont Auvergne, INSERM, U1240 Imagerie Moléculaire et Stratégies Théranostiques, Clermont-Ferrand, France; University of Bergen, NORWAY

## Abstract

It has been suggested that chemoresistance of chondrosarcoma (CHS), the cartilage tumor, is caused by the phenotypic microenvironmental features of the tumor tissue, mainly the chondrogenic extracellular matrix (ECM), and hypoxia. We developed and characterized a multicellular tumor spheroid (MCTS) of human chondrosarcoma HEMC-SS cells to gain insight into tumor cell biology and drug response. At Day 7, HEMC-SS spheroids exhibited a homogeneous distribution of proliferative Ki-67 positive cells, whereas in larger spheroids (Day 14 and Day 20), proliferation was mainly localized in the periphery. In the core of larger spheroids, apoptotic cells were evidenced by TUNEL assay, and hypoxia by pimonidazole staining. Interestingly, VEGF excretion, evidenced by ELISA on culture media, was detectable from Day 14 spheroids, and increased as the spheroids grew in size. HEMC-SS spheroids synthesized a chondrogenic extracellular matrix rich in glycosaminoglycans and type-2 collagen. Finally, we investigated the sensitivity of Day 7 and Day 14 chondrosarcoma MCTS to hypoxia-activated prodrug TH-302 and doxorubicin compared with their 2D counterparts. As expected, TH-302 exhibited higher cytotoxic activity on larger hypoxic spheroids (Day 14) than on non-hypoxic spheroids (Day 7), with multicellular resistance index (MCRI) values of 7.7 and 9.1 respectively. For doxorubicin, the larger-sized spheroids exhibited higher drug resistance (MCRI of 5.0 for Day 7 and 18.3 for Day 14 spheroids), possibly due to impeded drug penetration into the deep layer of spheroids, evidenced by its auto-fluorescence property. We have developed a model of human chondrosarcoma MCTS that combines an ECM rich in glycosaminoglycans with a high hypoxic core associated with VEGF excretion. This model could offer a more predictive *in vitro* chondrosarcoma system for screening drugs targeting tumor cells and their microenvironment.

## Introduction

The preclinical screening and validation process for new cancer drugs using 2D cell culture has been criticized for letting through many substances that go on to fail in clinical trials. The main causes of this attrition are unpredictable toxicity and/or low clinical efficacy [1,2]. Disappointing results for efficacy have been mainly blamed on the inadequacy and limitations of the 2D culture systems conventionally used for *in vitro* anticancer agent screening. One limitation is the absence of the cell-cell and cell-extracellular matrix (ECM) interactions that occur *in vivo* and are essential to tumor proliferation and response to therapy.

The marked effects that the microenvironment of a solid tumor can have on drug behavior are well documented, particularly as regards drug distribution (diffusion homogeneity and gradients) in the tumor, and hypoxia [3,4]. The intercellular and extracellular links, with a concomitant elevation in the interstitial pressure, also form a physical barrier to drug diffusion, contributing to drug resistance, an effect that is not manifest in monolayer cell culture [5,6]. Given the limitations of monolayer cultures and the complexity of the native tumor microenvironment, researchers have developed various 3D models that offer some of the features of solid tumor tissues, such as gradient distribution of chemical and biological factors, expression of pro-angiogenic and Multi Drug Resistance (MDR) proteins, and dynamic, reciprocal interactions between the tumor and its stroma [7].

Compared with conventional cell monolayers, the heterogeneous architecture of multicellular tumor spheroids (MCTS) has been described as more closely reproducing *in vivo* tumor architecture. This is particularly true for large MCTS (diameter > 200 μm), formed by spontaneous aggregation of cells in culture, which produce their own ECM and exhibit concentric arrangements of cells, with different proliferation phenotypes (from strongly proliferating cells at the periphery of the spheroid to a central necrotic core) [8–10].

The ECM-rich phenotype in 3D culture has been especially recognized as protecting the cells from cytotoxic activity and resistance acquisition, especially for chondrosarcoma (CHS), which is notoriously resistant to conventional chemo- and radiotherapy [3,11,12]. CHS, the second most common type of skeletal malignancy after osteosarcoma, with a wide range of clinical behaviors, remains a challenge for oncologists: the overall survival of patients with unresectable disease has not been improved in the last few decades [13].

It has been suggested that chemoresistance of CHS is caused by the phenotypic microenvironmental features of the tumor tissue, mainly chondrogenic ECM, and hypoxia, both possibly impeding the penetration of chemotherapeutic agents into the ECM and their diffusion to the tumor cells [14].

Because CHS response to therapy is strongly affected by microenvironmental factors, the use of 3D culture appears particularly appropriate for the simultaneously study of key cellular parameters that affect drug response and tumor cell biology.

Given the above, and the lack of efficacious therapies for CHS despite promising responses in 2D studies [15], we developed a 3D culture of human HEMC-SS chondrosarcoma cells using the non-adhered surface method, to gain insight into drug response and tumor cell biology of these tumors with proteoglycan-rich ECM. CHS spheroids were first studied for growth and structure by H&E staining and surface electron microscopy (SEM). At each stage of growth, the distribution of proliferative and apoptotic cells throughout spheroid sections at different stages of development was determined using Ki-67 immunohistochemistry and terminal deoxynucleotidyl transferase (TdT)-mediated dUTP nick-end labeling (TUNEL) assay respectively. Characterization of the spheroid microenvironment was focused on hypoxia, VEGF quantification, and extracellular matrix PG and type-2 collagen. Finally, we determined how 3D structure affected CHS cell chemosensitivity using doxorubicin and hypoxia-activated prodrug TH-302 in large and small spheroids, relative to their 2D counterparts.

## Materials and methods

### Monolayer and spheroid cultures of human chondrosarcoma HEMC-SS cell line

Human HEMC-SS CHS cell line was obtained from the European Collection of Authenticated Cell Cultures, and cultured in DMEM/F12 medium (Life Technologies, Carlsab, CA, USA) supplemented with 10% fetal calf serum (Dutscher) and 4 μg/mL gentamicin.

To generate HEMC-SS spheroids, cells were harvested from culture flasks by trypsinization, and seeded in 96-well non-adherent plates (Nunc) with 100 μL of 0.5% methylcellulose (Bio-Techne) diluted in the whole culture medium at a density of 1000 cells per well. Cultures were maintained in a 5% CO_2_ humidified environment at 37°C.

Spheroid doubling time was determined from exponential linear regression of cell counts from spheroids dissociated by incubation with trypsin-EDTA (Life Technologies) for 5 minutes at 37°C

### Scanning electron microscopy (SEM) analysis of spheroid structure

HEMC-SS spheroids (Day 14) were fixed with 1.6% glutaraldehyde in 0.2 M sodium cacodylate buffer (pH 7.4) overnight at 4°C, then washed three times with 0.2 M sodium cacodylate buffer (pH 7.4). For secondary fixation, spheroids were immersed in 1% osmium tetroxide in 0.2 M sodium cacodylate buffer (pH 7.4). Fixed spheroids were subsequently dehydrated with a graded ethanol series (25, 50, 75, 95, and 100%), and immersed in hexamethyldisilazane (HMDS) for 10 minutes (two times) at room temperature. The spheroids were freeze-dried until the HMDS had evaporated, mounted, coated with palladium gold, and examined under a scanning electron microscope (JEOL) at an acceleration voltage of 5 kV.

### Histology analysis of HEMC-SS spheroids

Day 7, Day 14 and Day 20 spheroids were fixed using 10% formalin, dehydrated with ethanol, and paraffin-embedded. Sections 5 μm thick were cut and adhered to poly-l-lysine-coated glass microscope slides. After deparaffinization and rehydration, slides were stained with hematoxylin-eosin (H&E) to examine spheroid morphologies, or with Alcian blue (pH 1) to evidence aggrecan.

### Immunofluorescence analysis of HEMC-SS spheroids

Pimonidazole was added to HEMC-SS spheroids (Day 7, Day 14 and Day 20) at a final concentration of 200 μM 2 h before fixation and paraffin-embedding. Sections 5 μm thick were cut and adhered to poly-l-lysine-coated glass microscope slides. After deparaffinization and rehydration, antigen retrieval was conducted by incubating the slides in boiling 10 mM citrate buffer (pH 6) with 0.05% Tween (v/v) (30 minutes) for Ki-67 and pimonidazole, or by treatment with proteinase K (20 μg/mL, 2 minutes) for collagen II. Endogenous peroxidase was quenched by incubating the slides for 30 minutes in 0.3% H_2_O_2_, and non-specific sites were blocked in 1% BSA/PBS for 1 h. Slides were then incubated with the primary antibodies: anti-pimonidazole mouse monoclonal (Hypoxiprobe, 1/50, 1 h, room temperature), anti-Ki-67 rabbit monoclonal (Abcam #15580, 1/2000, overnight, room temperature) or anti-collagen II rabbit polyclonal (Abcam #34712, 1/200, overnight, 4°C). Secondary biotinylated goat anti-mouse or anti-rabbit IgG1 antibodies (Vector Laboratories BA-1000, 1/500, 1 h) were used. Biotin was then complexed with streptavidin-coupled HRP (Vector Laboratories #SA-5004, 1/500, 30 minutes), and HRP was detected using tyramide signal amplification following the manufacturer’s instructions (TSA-Alexa488, Invitrogen). Emitting fluorescence was detected using Axioplan microscopy (Zeiss), and images were recorded with an AxioCam MRs5 camera (Zeiss).

### Detection of apoptotic cells in HEMC-SS spheroids by TUNEL assay

After deparaffinization, rehydration and antigen retrieval of 5 μm thick sections of Day 7, Day 14 and Day 20 spheroids, slides were incubated for 1.5 h at 37°C with a mix of 20 U/μL TdT (terminal deoxynucleotidyl transferase, Thermo Scientific), 1 mM biotin-11-dUTP (Thermo Scientific) and 1 mM ATP in TdT buffer. After washes, slides were incubated with streptavidin-HRP (Vector Laboratories, 1/500) at room temperature for 30 minutes. HRP was then detected with tyramide signal amplification following the manufacturer’s instructions (TSA-Alexa555, Invitrogen). Nuclei were counterstained with Hoechst 33342. Emitting fluorescence was detected using an Axioplan microscope (Zeiss), and images were recorded with an AxioCam MRs5 camera (Zeiss).

### VEGF assay by ELISA

VEGF was measured on spheroid medium at Day 7, Day 14 and Day 20 after cell seeding, using the human VEGF Quantikine ELISA Kit (R&D systems #DVE00). Optical density was measured using a Multiskan^™^ GO instrument (Thermo Scientific).

### GAG assay

Spheroids at Day 1, Day 7, Day 14 and Day 20 were sampled and digested for 24 h at 60°C in a 0.6 mg/mL papain (SIGMA) and 0.25 mg/mL dl-dithiothreitol (SIGMA) solution diluted in a phosphate/EDTA buffer (10 mM NaH_2_PO_4_, 10 mM Na_2_HPO_4_, 1 mM EDTA, pH 6.8). GAG assay was then carried out using a sulfated glycosaminoglycan assay kit (Blyscan^™^, Biocolor).

### Cytotoxicity experiments

After trypsinization, HEMC-SS cells were seeded in 96-well plates at a density of 20.10^3^ cells in 150 μL of the corresponding culture medium, and left to adhere overnight. For 3D cell cultures, Day 7 and Day 14 spheroids were used. Increasing concentrations of doxorubicin (Abmol) or TH-302 (Abmol) diluted in DMSO (maintaining final concentration at 0.5% v/v) were added. After plate incubation for 72 h in a 5% CO_2_ humidified environment at 37°C, culture media were removed, monolayer and spheroid cultures washed with PBS, and cell viability determined by AlamarBlue assay. For this purpose, 150 μL of 2% resazurin solution (stock solution at 1.25 mg/mL in PBS) diluted in MEM culture medium without phenol red (Gibco) were added to cell cultures, and plates were returned to the incubator. Fluorescence was measured with an excitation/emission wavelength of 630/590 nm on a Fluoroskan Ascent FLTM instrument (Labsystem). Cytotoxic activity was expressed as the drug concentration that inhibited cell viability by 50% (50% inhibiting concentration, IC_50_) determined using Prism software version 6.0. The multicellular resistance index (MCRI), which is the ratio of the IC_50_ cultured in spheroids at Day 7 and Day 14 to the IC_50_ cultured in a 2D system, was also calculated.

### Evaluation of doxorubicin penetration in HEMC-SS spheroids

We used the auto-fluorescence property of doxorubicin to determine whether the 3D culture of HEMC-SS cells limited the diffusion of drugs into the deep layer of spheroids.

Doxorubicin penetration on Day 7 and Day 14 spheroids was evaluated by fluorescent microscopy. To this end, spheroids were incubated with doxorubicin at a final concentration of 75 μM for 2 h at 37°C, washed with PBS, and paraffin-embedded. Sections 5 μm thick were then cut and adhered to poly-l-lysine-coated glass microscope slides. After deparaffinization and rehydration, emitting fluorescence was detected using an Axioplan microscope (Zeiss), and images were recorded with an AxioCam MRs5 camera (Zeiss).

### Fluorescent image processing

Image J (Fiji package) was used for analysis of Ki-67 expression and TUNEL assay on spheroid sections [16]. Each spheroid section in a captured image was divided up into rings, from periphery to center. The total fluorescence of each ring was measured and normalized to its area using the integrated density and measurement function of the ImageJ software.

### Statistical analyses

Statistical analyses were carried out using Prism software version 6.0. Data are represented as means ± SD, and statistical significance was determined using a one-way ANOVA followed by a Tukey multiple-comparison post-test. Results were considered significant at *p* < 0.05 (*p < 0.05, **p < 0.01, ***p < 0.001).

## Results

### Spheroid growth

In this study, we used the crowding agent methylcellulose, a cellulose-derived inert compound that helps cells aggregate and form spheroids. After testing various starting cell numbers per well (250 to 2000 cells) and different concentration of methylcellulose (from 1% to 0.25%) (data not shown), we found that 1000 cells per well in 0.5% of methylcellulose was optimal for a 20-day study period. In these conditions, HEMC-SS cells formed one centrally positioned spheroid in each well of the conical bottom of non-adherent 96-well plates. The spheroids were cultured for 5 days before the first medium change to allow the formation of ECM.

**Fig 1:**
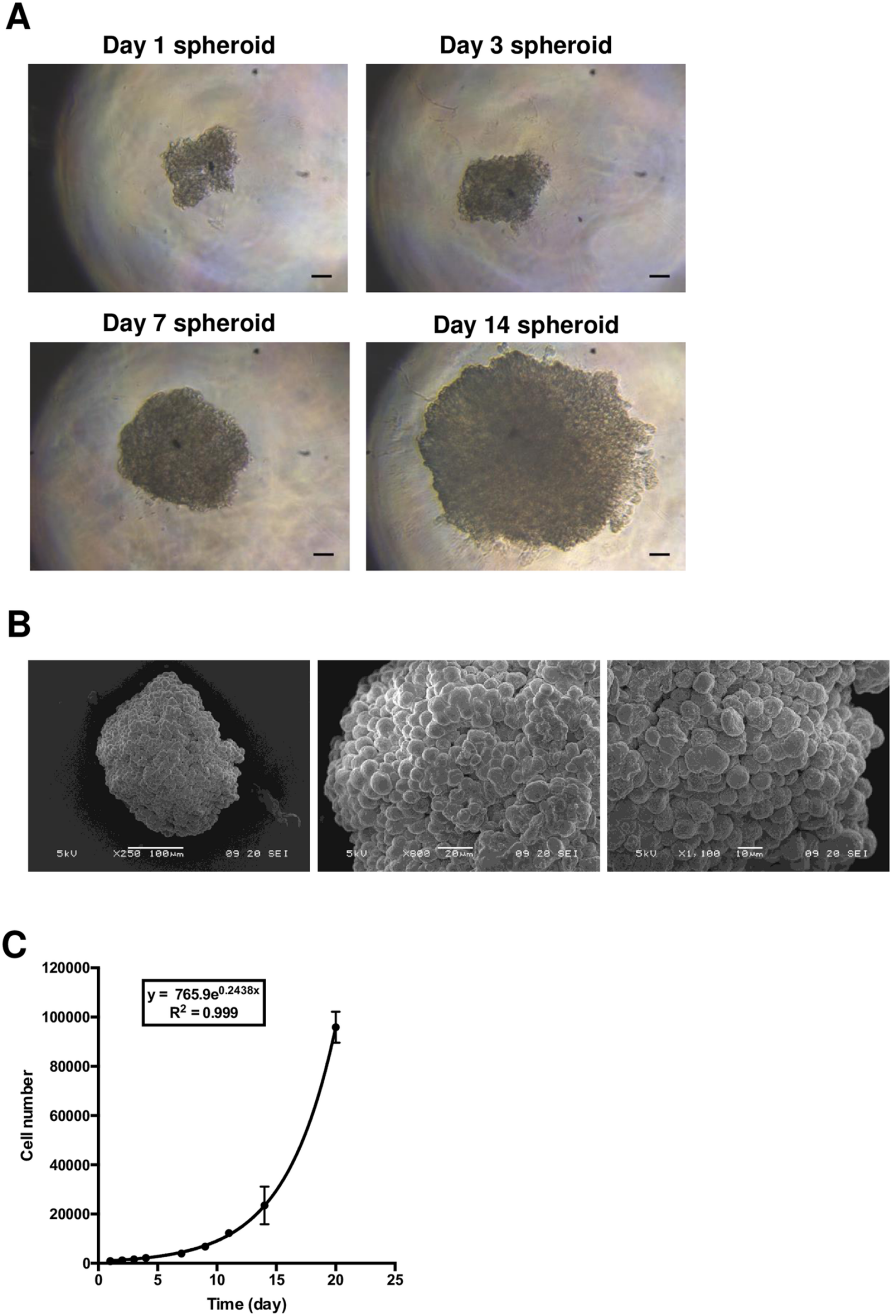
Formation of HEMC-SS spheroids. To generate spheroids, 1000 human chondrosarcoma HEMC-SS cells were seeded in a non-adherent 96-well plate in a solution of 0.5% methylcellulose diluted in corresponding culture medium. (A) Optical visualization of HEMC-SS spheroids at different stages of development. Scale bar = 100 μm (B) SEM imaging of one representative spheroid at Day 14. (C) Cell count of trypsin-dissociated spheroids.

Culturing of human cell line according to the 3D protocol showed that HEMC-SS cells aggregated from Day 1 post-seeding of 1000 cells (Fig 1A). In the initial stage, single cells spontaneously self-assembled to form cell aggregates in each micro-well, and individual cells could be easily identified (Day 1). From Day 7, multicellular aggregates formed round spheroids with well-defined contours and continuous surfaces. Scanning electron microscope examination of HEMC-SS spheroids on Day 14 confirmed the formation of a three-dimensional spherical architecture (Fig 1B).

The growth kinetics of HEMC-SS spheroids was assessed longitudinally by cell counting (Fig 1C). Exponential growth was observed with a cell count of 915 ± 202 at Day 2 after cell seeding, and 95833 ± 6331 at Day 21. The spheroid doubling time, determined from the exponential regression of cell number curves, was 2.79 ± 0.05 days (2.1 days for 2D cells).

### Characterization of chondrosarcoma spheroid structures

**Fig 2:**
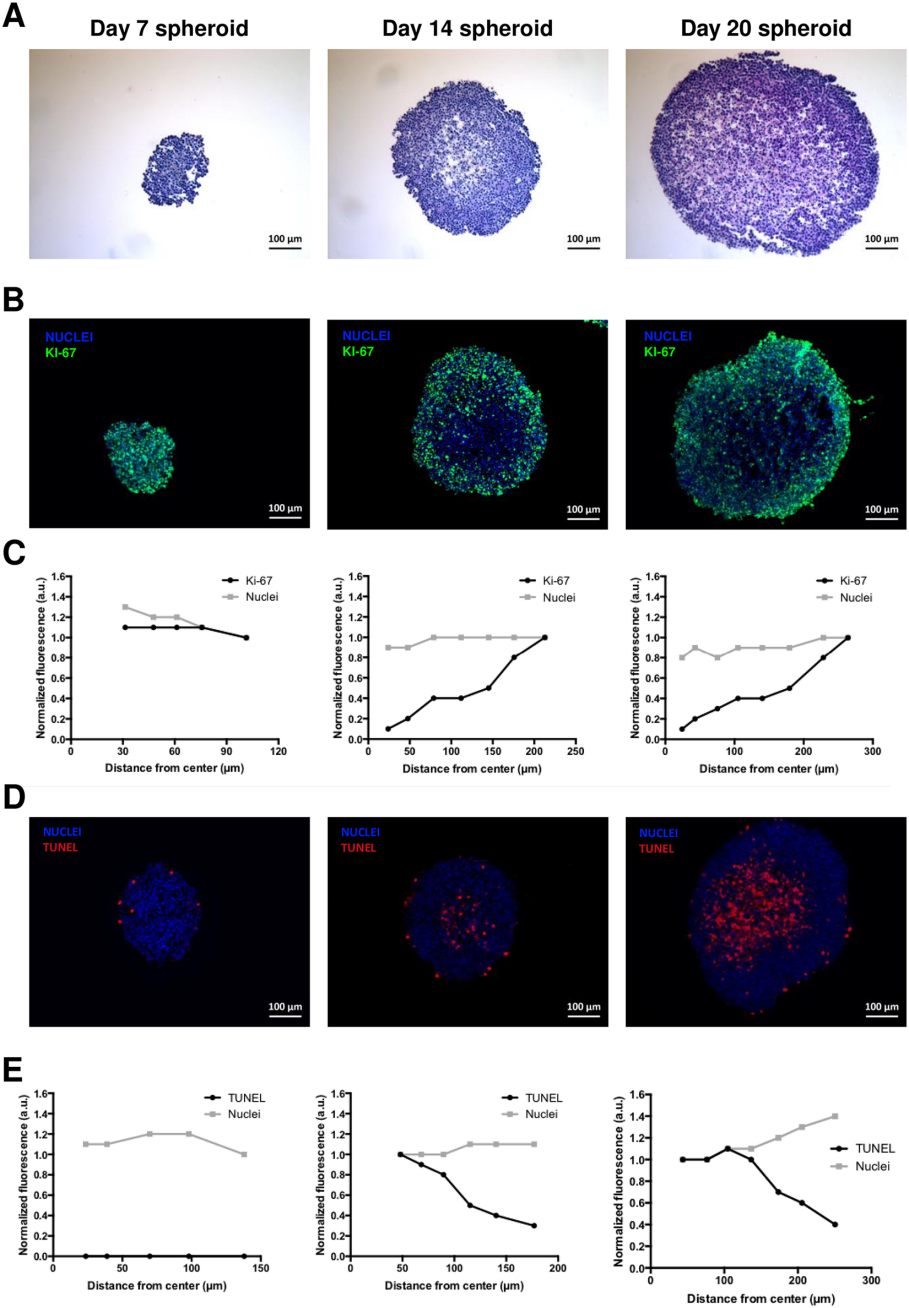
(A) H&E staining of spheroid sections. (B) Determination of proliferative cells on spheroid sections by immunohistochemistry using the proliferation marker Ki-67 (green). Blue represents nuclei staining (C) Analysis of stained sections was used to compare the distribution of nuclei and proliferative cells throughout each section. (D) TUNEL assay (red) on spheroid sections to detect apoptotic cells. Blue represents nuclei staining (E) Analysis of stained sections was used to compare the distribution of nuclei and apoptotic cells throughout each section.

The morphology of spheroids according to the stage of development was also assessed by H&E staining of spheroid sections (Fig 2A). At Day 7, H&E staining was found to be homogeneously distributed, but from Day 14, a peripheral area with a highly compact, dense cellular network stood out from the more diffuse central one. This feature was more pronounced for Day 20 spheroids.

### Evaluation of proliferative and apoptotic cells in chondrosarcoma spheroids

Immunofluorescence studies with the proliferation marker Ki-67 and TUNEL assay were conducted on spheroid sections. Both staining methods showed differential distributions of proliferative and apoptotic cells, as illustrated in Fig 2B and 2D, with corresponding quantitative analysis presented in Fig 2C and 2E respectively. Spheroids at Day 7 showed proliferative cells distributed homogenously throughout the section, while in Day 14 and Day 20 spheroids they were mainly peripherally located (Fig 2B). The quantitative analysis of stained sections to compare the distribution of Ki-67 (green) and nuclei (blue) throughout the spheroid sections confirmed these observations: from Day 14, the signal profile of Ki-67 positive cells decreased toward the center of the spheroids (Fig 2C), while the proportion of nuclei remained constant throughout the section. TUNEL assay detected apoptotic cells mainly in the center of spheroids at Day 14 and Day 20. Fig 2D clearly shows that apoptotic cells were highly concentrated in the center of the spheroids, and decreased toward the periphery as early as Day 14 (Fig 2D and 2E). The proportion of apoptotic cells was greater in the spheroids at Day 20.

### Evaluation of hypoxia distribution in chondrosarcoma spheroids

**Fig 3:**
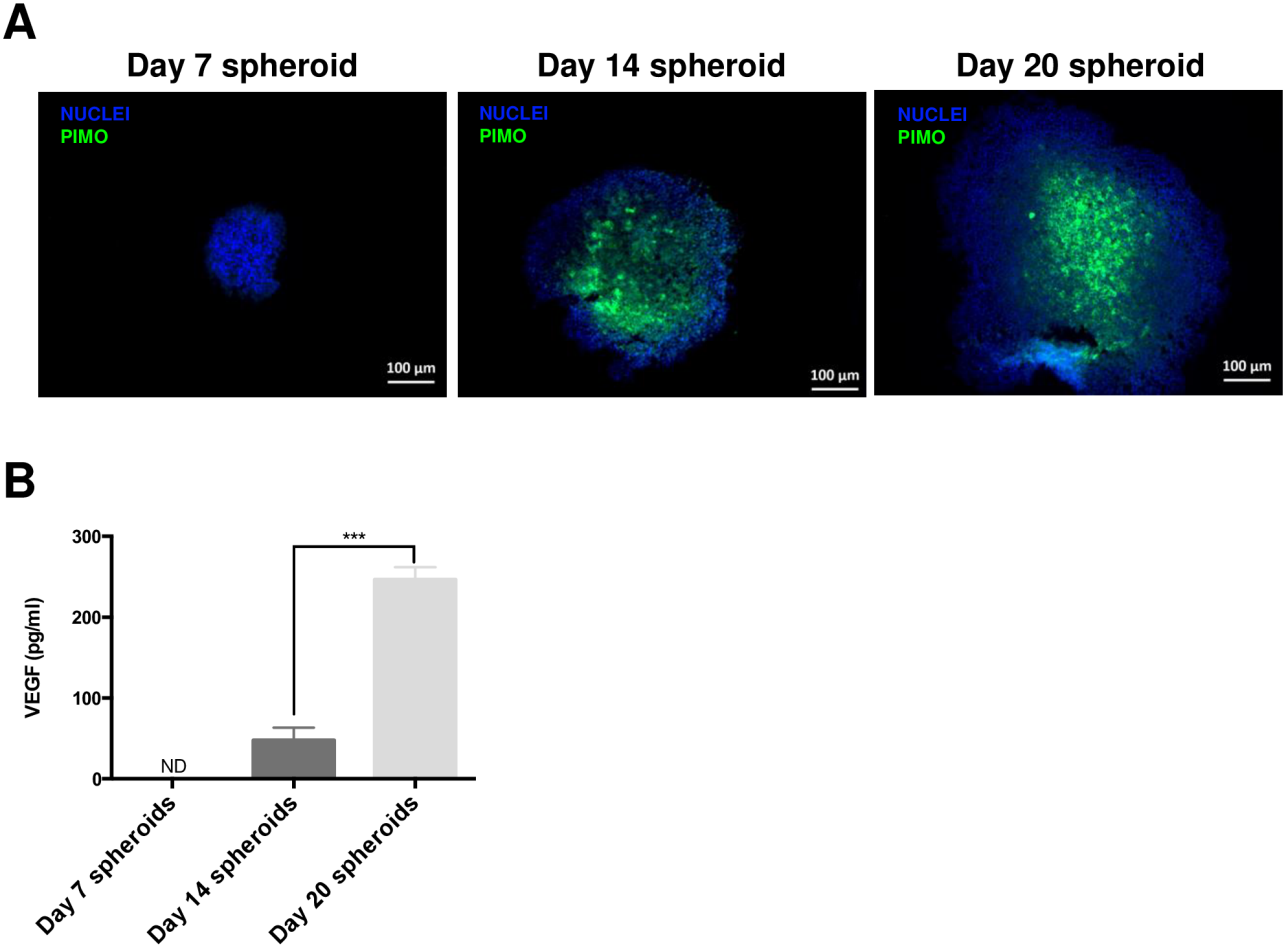
Hypoxia determination in HEMC-SS spheroids. (A) Hypoxia was assessed by pimonidazole staining of spheroid sections: 200 μM final concentration of pimonidazole was added to spheroid supernatants 2 h before embedding spheroids in paraffin. (B) Quantification of excreted VEGF in spheroid medium by ELISA.

We went on to determine whether hypoxia could explain the lack of proliferation and the presence of apoptotic cells in the core of large spheroids (Day 14 and Day 20). For this purpose, we used a 2-nitroimidazole probe, pimonidazole, which covalently binds to thiol groups of molecules in hypoxic tissues with an oxygen partial pressure lower than 10 mmHg. At Day 7, spheroids were not stained by pimonidazole, whereas sections of spheroids at Day 14 and Day 20 were stained positively in their core regions (Fig 3A).

### VEGF excretion by spheroids

Since tumor cells are known to excrete VEGF in response to a hypoxic environment *in vivo*, we investigated VEGF excretion as chondrosarcoma spheroids grew in size and developed hypoxia: VEGF excretion, evidence by ELISA on culture media, was detectable from Day 14 spheroids with a value of 10.5 ± 4.2 pg/mL, and increased as the spheroid grew in size (Fig 3B). At Day 20, the increase in VEGF excreted was significant (*p* < 0.001) relative to Day 14 spheroids.

### Evaluation of GAG and collagen in ECM of chondrosarcoma spheroids

We investigated the distribution of two major constituents of the chondrosarcoma extracellular matrix, GAG of proteoglycans, and type-2 collagen.

**Fig 4:**
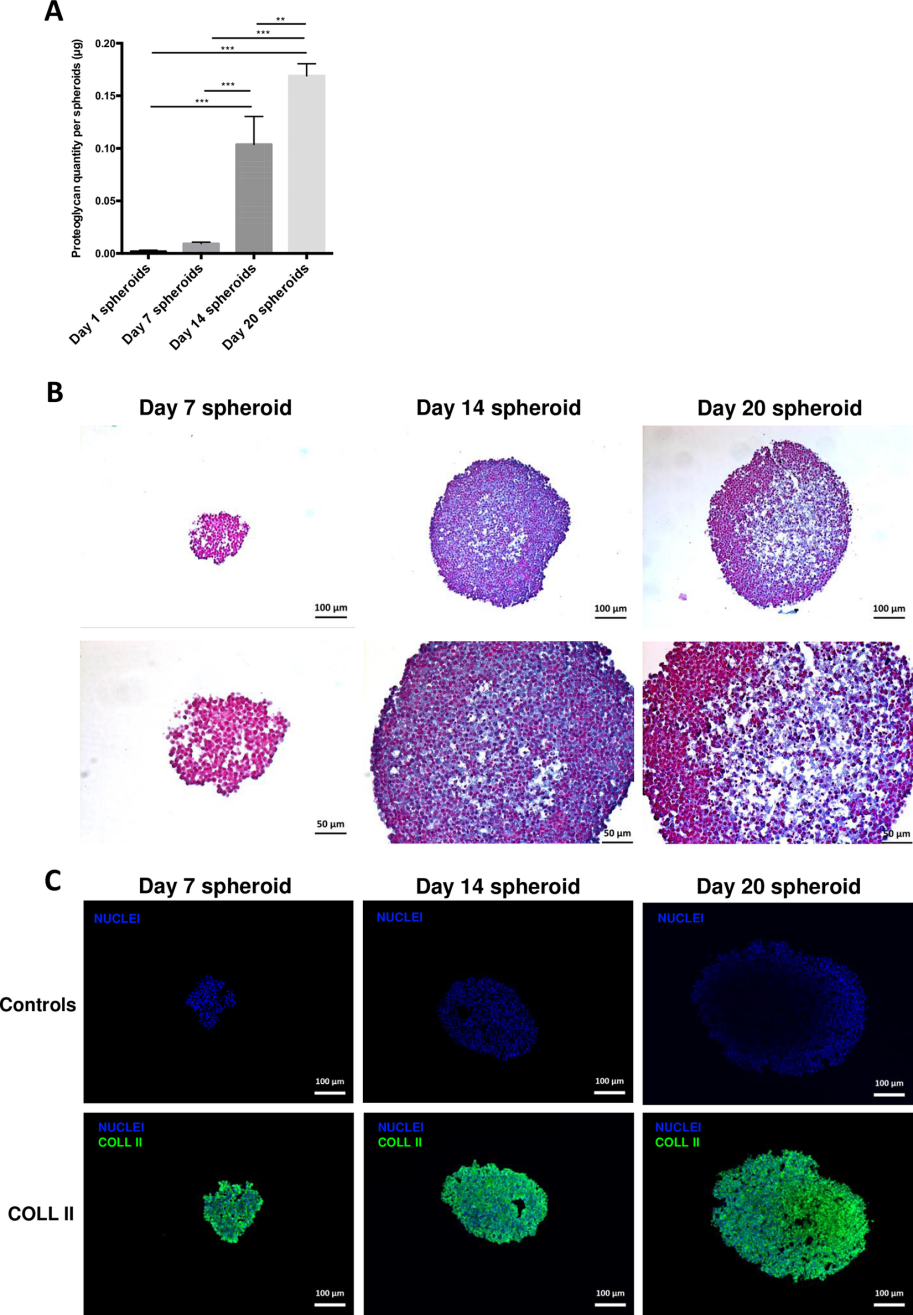
(A) GAG quantification in spheroids using sulfated glycosaminoglycan assay kit. (B) Alcian blue staining of Day 7, Day 14 and Day 20 spheroid sections. (C) Immunofluorescence of type-2 collagen (green) performed on Day 7, Day 14 and Day 20 spheroid sections. Blue represents nuclei staining. Controls were obtained by omitting primary antibody but using comparative illumination parameters.

Firstly, Blyscan assay on spheroids at Day 1, Day 7, Day 14, and Day 20 demonstrated that GAG amount increased as the spheroids grew in size, probably owing to a greater number of producing cells (Fig 4A and S1 Fig). GAG distribution within the spheroid sections was determined by Alcian blue staining carried out on Day 7, Day 14 and Day 20 spheroids (Fig 4B). No staining was observed for Day 7 spheroids, but Alcian blue was homogeneously distributed within the spheroid section at Day 14, and was mainly localized in the center of Day 20 spheroids. Expression of type-2 collagen, determined by immunofluorescence, highlighted a homogeneous distribution of type-2 collagen throughout the sections of Day 7, Day 14 and Day 20 HEMC-SS spheroid sections (Fig 4C). These results confirmed that HEMC-SS spheroids synthesize a chondrogenic extracellular matrix.

### Drug cytotoxicity assay

**Fig 5:**
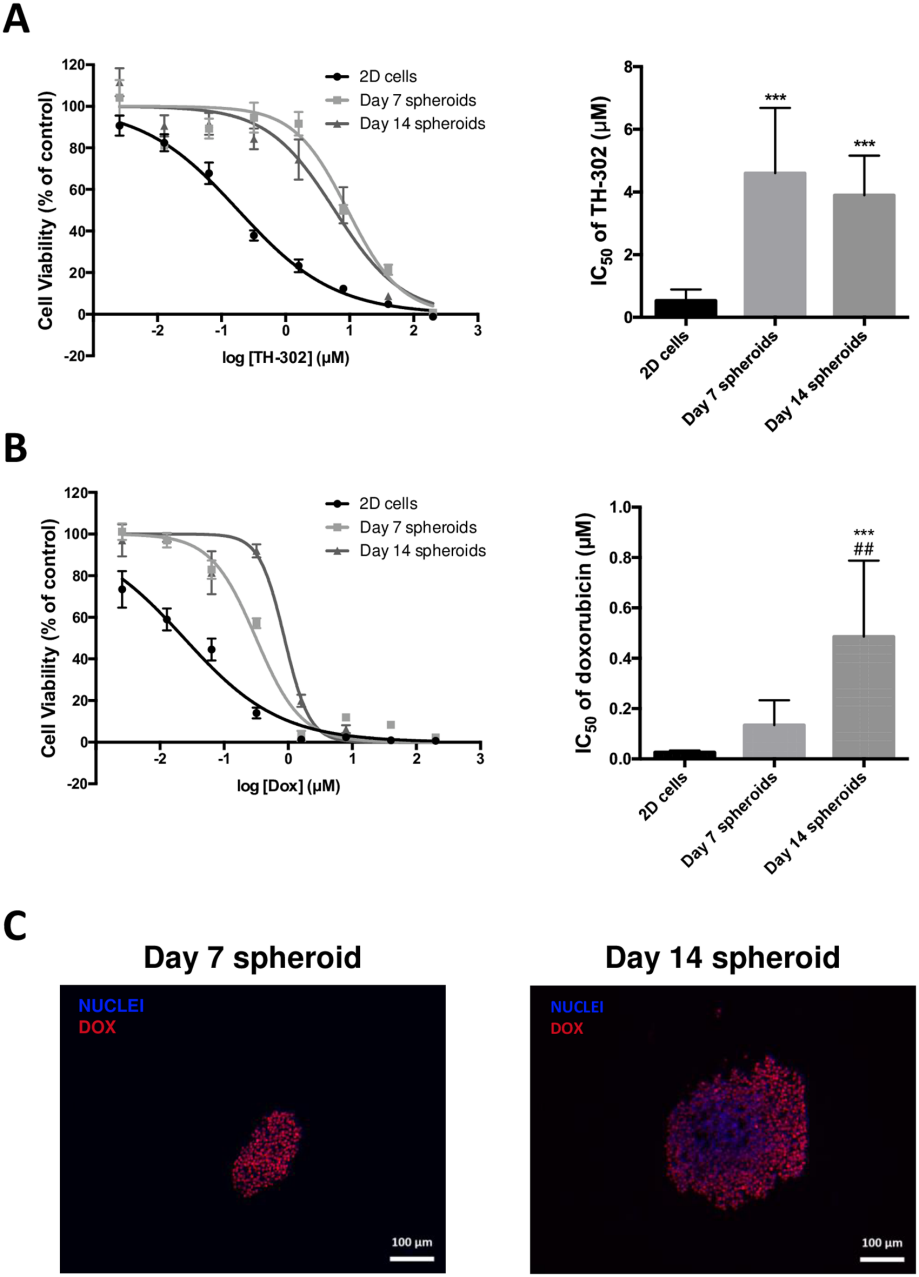
Assessment of cytotoxicity of TH-302 (A) and doxorubicin (B) on Day 7 and Day 14 spheroids relative to cells cultured in a monolayer. Cultures were exposed to drugs for 72 h. * significant difference versus 2D cells; # significant difference versus Day 7 spheroids. (C) Doxorubicin penetration on Day 7 and Day 14 spheroid sections evaluated by fluorescent microscopy; spheroids were incubated with doxorubicin at a final concentration of 75 μM for 2 h.

AlamarBlue cell viability assays were performed to determine drug cytotoxicities of conventional chemotherapy (doxorubicin) and the hypoxia-activated prodrug TH-302 on HEMC-SS spheroids at Day 7 and Day 14, and compared with monolayers. IC_50_ and multicellular resistance index (MCRI) values are summarized in Fig 5A and 5B and Table 1.

**Table 1:** IC_50_ (μM) and multicellular resistance index (MCRI) values of culture for drug resistance evaluation.

	TH-302		Doxorubicin	
	IC_50_ (μM)	MCRI	IC_50_ (μM)	MCRI
**2D cells**	0.505 ± 0.313	/	0.026 ± 0.007	/
**Day 7 spheroids**	4.602 ± 2.087	9.1	0.134 ± 0.010	5.0
**Day 14 spheroids**	3.894 ± 1.268	7.7	0.485 ± 0.302	18.3

For the hypoxia-activated prodrug TH-302, higher cytotoxic activity was evidenced on larger hypoxic spheroids (Day 14), with an MCRI of 7.7 compared with smaller normoxic spheroids (Day 7) with an MCRI of 9.1.

For doxorubicin, spheroids displayed a significant higher drug resistance as they grew in size compared with their 2D counterparts (Fig 5B). The MCRI was 18.3 at Day 14 versus 5.0 at Day 7 (Table 1). To determine whether resistance could be attributed to doxorubicin penetration in spheroids, we measured its auto-fluorescent signal in Day 7 and Day 14 spheroid sections after 2 h of incubation (Fig 5C). On Day 7 spheroids, the doxorubicin signal was homogeneously distributed throughout the section, while on Day 14 spheroids it was restricted to the outer few cell layers. These results suggest that the ability of doxorubicin to penetrate spheroids depends on their size and possibly on the cause of the resistance.

## Discussion

Three-dimensional cultures of tumor cells have recently gained attention for their greater ability to predict behavior of new drugs in preclinical and clinical trials, compared with 2D cultures, which lack stroma and cellular heterogeneity, and cannot reproduce transport gradients [17].

This applies particularly to CHS, which is notoriously highly resistant to most cytotoxic drugs, mainly owing to cellular heterogeneity, hypoxia, and a chondrogenic ECM limiting drug diffusion [15,18]. It has been reported that many tumor cells are able to form aggregates instead of well-defined spheroids [17,19]: we therefore investigated the behavior and phenotypic features of human chondrosarcoma cells in 3D culture.

Many methods have been described for obtaining spheroids, such as suspension culture, the non-adherent surface method, hanging drop method, microfluidic method, and pellets [20]. For chondrosarcoma cells, the main methods published are pellets, which use centrifugal force to favor cell-to-cell adhesion at the bottom of a tube [21]. Although this method is simple and rapidly implemented, it may generate shear stress in the cells [22]. We therefore opted for the non-adherent method with the use of methylcellulose, which increases the viscosity of media, and enables spontaneous aggregation of cells in stationary culture, which can thus produce their own ECM [9,23].

HEMC-SS MCTS culture demonstrated homogeneous size and growth, and high reproducibility. From Day 14 after cell seeding, large spheroids exhibited a heterogeneous architecture with a peripheral proliferating region and an apoptotic core. From Day 14, Ki-67 positive cells decreased in number toward the center of spheroids, while TUNEL positive cells decreased in number toward the periphery. This observation was consistent with published data showing that larger spheroids (radius > 200–300 μm) developed central necrosis when cells died by apoptosis or necrosis [24,25]. Given the average radius of our large spheroids (250 ± 7 μm at Day 14), we hypothesized that the limited diffusion of nutrients and oxygen to cells localized in the central region inhibited their proliferative activity, consistent with the suggested free diffusion distance of 150–200 μm [26].

Tumor hypoxia has been implicated in processes including cancer cell survival, resistance to cell death, tumor angiogenesis, invasion, metastasis, radioresistance and chemoresistance [26]. Hypoxic regions are well recognized in CHS, and are associated with poor prognosis [27,28]. In CHS 3D culture, hypoxic areas, evidenced by pimonidazole staining, were localized in the core of large spheroids (Day 14 and Day 20). Interestingly, in response to hypoxic stress, large spheroids (Day 20) excreted pro-angiogenic VEGF factor. By contrast, small spheroids (Day 7) unstained by pimonidazole did not excrete VEGF. These results are in line with a recent published study demonstrating that VEGF is excreted on the outside of the spheroid *in vitro* and upregulated by hypoxia [29]. In addition, chondrosarcoma spheroids have been demonstrated to produce a chondrogenic extracellular matrix rich in GAG and type-2 collagen.

We investigated whether 3D culture of CHS cells mediated drug resistance. To our knowledge this is the first study of cell behavior and sensitivity to drugs of human chondrosarcoma MCTS versus their 2D counterparts. Using homogeneous spheroids of two different sizes, we assessed drug cytotoxicity dependence, and compared the results with those from 2D cultures. For this purpose, we analyzed the cytotoxic effect of two drugs with two different mechanisms of action and molecular physical and chemical properties: the intercalating agent doxorubicin, and the hypoxia-activated prodrug TH-302, which had already been tested on primary bone tumor and moved on to clinical trials [30–34]. Both drugs demonstrated cytotoxic potency in CHS cells, with IC_50_ values in the micromolar range. For doxorubicin, 3D culture of HEMC-SS cells induced resistance relative to 2D culture. We used the auto-fluorescence property of doxorubicin to determine whether resistance was due to a limited penetration into the deeper layers of spheroids. As expected, on Day 14 spheroids, doxorubicin was restricted to the outer few cell layers. These results were consistent with previously published data showing that doxorubicin effectiveness was inversely proportional to distance from the MCTS periphery [10,35].

Like hypoxic areas in tumors, the oxygen gradation in MCTs provides a hypoxic core that could also be responsible for reducing drug efficacy, chemotherapeutics exhibiting limited toxicity in oxygen-deprived and acidic tumor microenvironments. From our results, large hypoxic spheroids (Day 14) were more resistant to doxorubicin (MCRI = 18.3) than to TH-302 (MCRI = 7.7).

Interestingly, when spheroids grew in size and exhibited large hypoxic cores at Day 14, their cells were more sensitive to TH-302 (MCRI = 7.7) than those of smaller hypoxic spheroids at Day 7 with MCRI = 9.1.

We consider that the sensitivity of chondrosarcoma cells to TH-302 in 2D cultures, where no hypoxia occurs, could be attributed to the cytotoxicity of superoxide species, generated under normoxia for a 72 h incubation time [36]. This phenomenon had previously been observed by Mang et al., who reported that under normoxia, TH-302 cytotoxicity in H460 cells increased with drug exposure time (IC_50_ after 1 h = 94 μM versus IC_50_ after 24 h = 3.3 μM) [37].

It has also been postulated that the drug resistance observed could additionally result from the cell adhesion between tumor and stroma cells that occurs in the 3D systems [38,39], and from the presence of various layers with cells in different stages of the cell cycle [35].

Understanding the importance of cell adhesion and ECM may lead to the identification of new targets in the stroma, including stromal cells, ECM entities, matrix-degrading proteases and inhibitors, and regulatory substances [40]. The 3D culture of HEMC-SS cells as MCTS developed in this study can yield reliable information on the relationship between tumor cells and their microenvironment, through intercellular and extracellular links, highlighting the role of ECM in cartilaginous tumor biology and its importance in regard to therapy.

## Conclusion

To our knowledge, this study is the first to demonstrate that multicellular tumor spheroids of human HEMC-SS cells combine two favorable attributes: (i) a chondrogenic extracellular matrix rich in GAG and type-2 collagen, and (ii) a high hypoxic core from Day 14 of growth associated with VEGF excretion. Although 3D cell culture can never totally recreate the *in vivo* system, its features argue in favor of chondrosarcoma MCTS as a powerful alternative for drug screening, and could facilitate the emergence of targeted, personalized approaches to unresectable chondrosarcoma.

## Supporting information

S1 Fig GAG amount normalized by spheroid volume at day 1, day 7, day 14. Spheroid volumes were determined by measuring area (S) of the 2D projection of the spheroids to calculated the radius (R = √(S/π)) and the volume (V = 4/3 π R^3^) of an equivalent sphere.
(TIF)
