# Supplementary figures and images for: Development and characterization of a human three-dimensional chondrosarcoma culture for in vitro drug testing
Source: PLoS One. 2017 Jul 13;12(7):e0181340. doi: 10.1371/journal.pone.0181340 (PMC5509331; doi:10.1371/journal.pone.0181340)

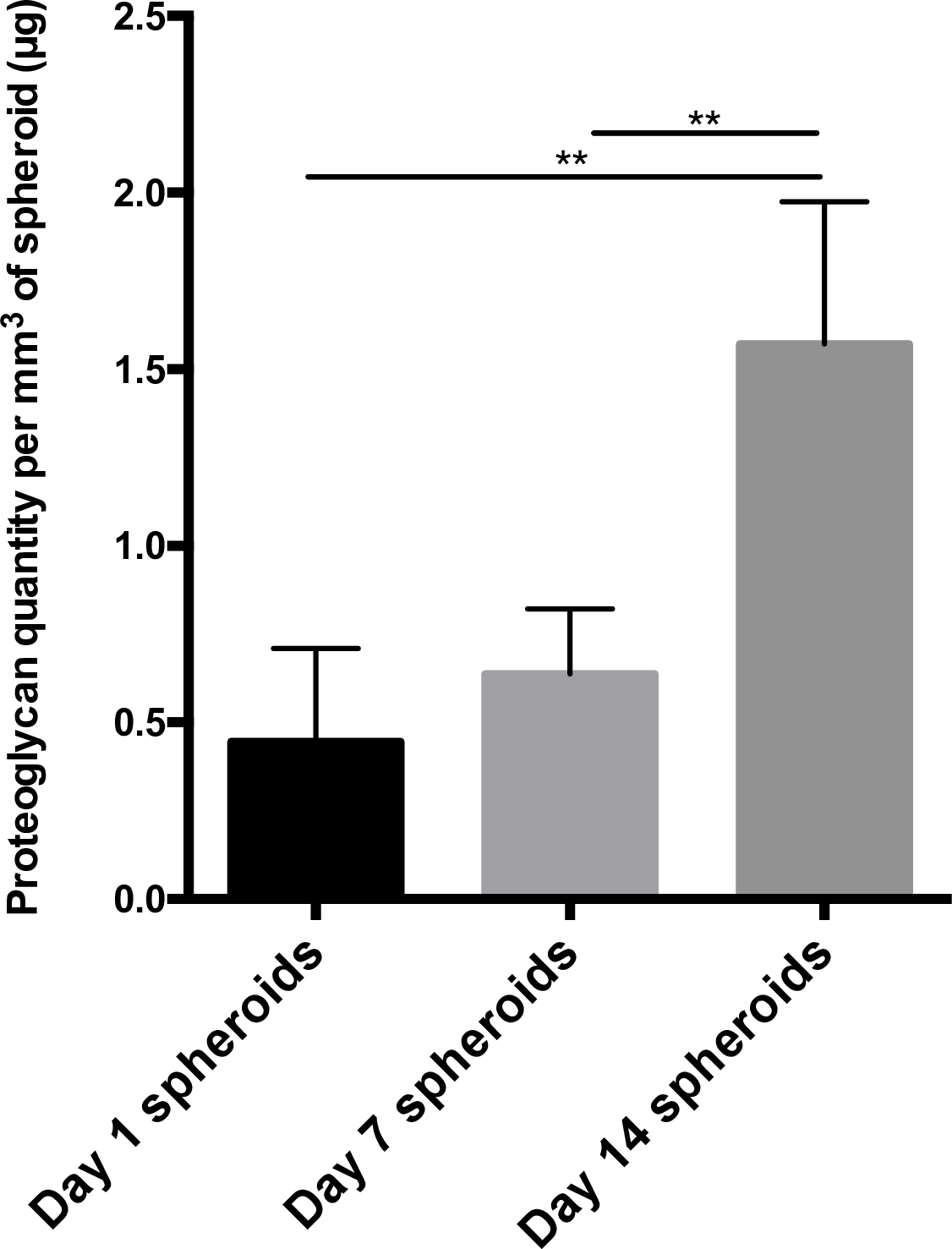

Supplement: S1 Fig — Spheroid volumes were determined by measuring area (S) of the 2D projection of the spheroids to calculated the radius (R = √(S/π)) and the volume (V = 4/3 π R3) of an equivalent sphere. (TIF) [file pone.0181340.s001.tif]
